# Two-thirds of older people is interested in information meetings on end-of life care to stimulate advance care planning: a national survey

**DOI:** 10.1186/s12877-025-06231-x

**Published:** 2025-07-31

**Authors:** Tessa D. Bergman, Annicka G. M. van der Plas, Bregje D. Onwuteaka-Philipsen, H. Roeline W. Pasman

**Affiliations:** Department of Public and Occupational Health, Amsterdam Public Health Research Institute, Expertise Centre for Palliative Care, Amsterdam UMC, Vrije Universiteit Amsterdam, P.O. Box 7057, Amsterdam, 1007 MB The Netherlands

**Keywords:** Palliative care, End-of-Life care, Community, Information meetings, Older people, Advance care planning, Cross-Sectional study

## Abstract

**Background:**

Information meetings are a way to create awareness and inform older people on end-of-life care and have shown to stimulate advance care planning (ACP). Before further development and implementation, it is important to explore older people’s preferences regarding these meetings. This study aims to explore which older people are interested, whether this is associated with previous ACP behaviour, and their preferences regarding the content and organisation of information meetings.

**Methods:**

A cross-sectional study was conducted on a randomly drawn sample of 1242 Dutch older people (≥ 65 years; response 93.2%) from a nationwide panel. In September 2020, they received a questionnaire on palliative care, including questions on interest in attending an information meeting. Descriptive statistics, chi-square tests, and regression analyses were performed.

**Results:**

One-third of respondents reported they would attend an information meeting if they were invited this week (33.5%), 33.2% (possibly) in the future and 33.3% would not attend. Respondents who had thought about end-of-life topics (OR 2.54 CI 1.63–3.96) were more likely to be currently and (possibly) interested in the future in attending an information meeting compared to those who had not. Main topics of interest included possibilities for care at home (88.1%), symptom relief (87.7%), and advance directives (80.6%). Further, respondents preferred to be invited through a personal invitation from their general practitioner (51.2%) or another organisation such as the municipality (54.7%). Also, they preferred their own general practitioner (48.3%) or another healthcare facility such as a hospital or care home (17.9%) to organise information meetings.

**Conclusions:**

This study shows that information meetings on end-of-life care are appealing to a large and diverse group of older people. Information meetings should cover a broad range of topics, ranging from possibilities of care at home and symptom relief to end-of-life decisions. For these meetings, older people prefer to be invited through a personal letter and value the involvement of their general practitioner. Therefore, meetings should preferably be organised by or in collaboration with the general practitioner.

**Supplementary Information:**

The online version contains supplementary material available at 10.1186/s12877-025-06231-x.

## Introduction

*“Do you want to be resuscitated?” asked the paramedic when the 83-year-old man was getting into the ambulance with chest pain.* Do I want to be resuscitated? *“Why wouldn’t I want to be resuscitated?”* Old age comes with declining health; older people are more likely to be frail, have more comorbidities due to aging, and will increasingly depend on healthcare [1]. It is expected that older people have to make vital choices regarding care in the near future and may need to shift their goals of care [2]. Therefore, advance care planning (ACP) may be especially relevant for them. ACP enables individuals to define goals and preferences for future medical treatment and care, to discuss these goals and preferences with family and healthcare professionals, and to record and review these preferences if appropriate [3]. Ideally, ACP is an ongoing process, and not a one-time decision [4].

Currently ACP is often not initiated until people are diagnosed with a life-limiting disease [5]. However, it has been argued that ACP conversations should start when people are in good health and living independently in the community [6]. Research among older people suggests that the preference to first independently conceptualize wishes and preferences for future care [7] and lack of knowledge of ACP and end-of-life decisions [8] are barriers to ACP. In addition, our previous study showed that knowledge of palliative care is limited in Dutch older people, possibly hindering engagement in and success of ACP [9]. Further, lack of time prevents healthcare professionals from addressing ACP with older people [10, 11], which is also recognized by older people [12].

Several studies have shown that community interventions with educational purposes about ACP and/or end-of-life care (i.e. workshops, group visits, information meetings) stimulate ACP in the general public [13–15], and more specifically in older people [6, 16–20]. These interventions may also overcome time constraints of healthcare professionals as (general) information is provided in a group setting instead of individually. In order to further develop and implement information meetings on end-of-life care on a larger scale and in different settings, it is important to explore what older people’s preferences are for these meetings. To our knowledge, this is still unknown. Therefore, this study aims to explore I) how many and which older people are interested in attending information meetings and II) what older people prefer regarding content and organisation of information meetings on end-of-life care.

## Methods

### Study design and data collection

A cross-sectional survey study was conducted with the use of data of the Longitudinal Internet Studies for the Social sciences (LISS) administered by Centerdata (Tilburg University, The Netherlands). The LISS panel is a probability-based sample of Dutch individuals. When starting the panel, 9,842 households were approached of which 4,725 households participated with one or more household member (48%). This resulted in 7,756 individuals participating in the panel [21]. Extensive effort was put into ensuring representativeness of the sample (including providing internet access for households without internet and simple devices for people with limited digital skills) [21], resulting in similar representativeness of the LISS-panel to traditional surveys based on probability sampling [22, 23]. Refreshment samples were included to correct for initial selection biases and continued for correction of possible attrition bias [24].

### Ethics and informed consent

During the recruitment of the panel, respondents who agreed to participate in the panel received a confirmation email, and a letter with login code. With the login code provided they can confirm their willingness to participate and immediately start the first questionnaire. At the end of this questionnaire, respondents are asked to read and agree to the LISS informed consent. Only when respondents agree to the LISS informed consent they can become a LISS panel member. This confirmation and informed consent procedure, following the consent to participate given to the interviewer, ensures the double consent of each respondent to become a panel member [25].

### Study population

The LISS panel participates in a yearly longitudinal questionnaire, covering a large variety of domains including demographics [21, 26], and monthly questionnaires on specific topics. In September 2020, a questionnaire about palliative care, specifically designed for this study by Amsterdam University Medical Center (Amsterdam UMC), was send to a randomly drawn sample of 1,333 individuals aged 65 and older from the LISS panel. This resulted in a response rate of 93.2% (*N* = 1,242).

### Measurements

#### Demographics

Demographic characteristics were obtained from the yearly questionnaire and included: age (65–70/70–75/75–80/≥80), sex (male/female), migration background (yes/no), religiosity (yes/no), level of education (low/middle/high), net household income (below basic state pension (AOW): ≤1,350 euro/between basic state pension and median income: 1,350-2,350 euro/between median income and 1.5 times median income: 2,350-3,850 euro/more than 1.5 times median income: >3,850 euro) and urbanisation (very urban/strongly urban/moderately urban/not very urban/not urban). Second, health- and care-related characteristics included: self-perceived health status (good/bad), health literacy (adequate/inadequate) according to Chew’s Set of Brief Screening Questions [27], experience with palliative care through family, friends and/or acquaintances (yes/no), frequency of contact with the general practitioner (GP) and medical specialist (MS) per year (none/1–4 times/5 or more), and trust in the GP providing good care at the end of life and respecting wishes at the end of life (no to not much trust/some to a lot of trust). Questions on demographics are in accordance with how Statistic Netherlands uses them [28]. Measurements are described extensively in Appendix 1.

#### Advance care planning behaviour

Respondents were asked whether they had thought about, discussed with family/friends, discussed with a healthcare professional, and/or had written down wishes about the following topics: whether or not they want/can continue to live at home, would like to go to hospital, would like to be admitted to a nursing home, want to be resuscitated, which treatments they would and would not want any more in certain circumstances, would want euthanasia in certain circumstances, and who could make medical decisions for them if they are no longer able to do so themselves. First, respondents were categorised in the following (mutually exclusive) categories: people who had and had not thought about end-of-life topics. People who had thought about end-of-life care were further categorised in the following (not mutually exclusive) categories of ACP behaviour: people who [1] discussed end-of-life topics with family/friends, [2] discussed end-of-life topics with a healthcare professional, and [3] had written down wishes regarding end-of-life care (advance directive).

#### Interest in attending an information meeting

Respondents were asked I) ‘Suppose you receive an invitation for an information meeting on end-of-life care *this week*. Would you go?’ (yes/no). When respondents answered ‘yes’, they were asked why: (a) the subject is important to me, (b) the subject is important to loved ones, (c) I want to know more about the end of life, and/or (d) I am thinking about the end of life (multiple answers possible). When respondents answered ‘no’, they were asked II) ‘Do you think you would attend an information meeting on end-of-life care *in the future*? (yes, definitely/yes, maybe/no). Also, they were asked why they were not interested in attending an information meeting (open-ended question). For the purpose of analyses, the two questions on interest were categorized into [1] currently interested (answered ‘yes’ to the first question), [2] (possibly) interested in the future (answered ‘yes, definitely’ or ‘yes, maybe’ to the second question) and [3] not interested (answered ‘no’ to the first and second question). The categories ‘currently interested’ and ‘(possibly) interested in the future’ were analysed separately as well as together.

### Statistical analysis

Statistical analyses were performed using SPSS IBM 28. Descriptive statistics and chi-square tests were used to assess respondents' characteristics and their interest in information meetings, and to explore what topics respondents were interested in, from whom they wanted to receive information and an invitation, and who they preferred to organise information meetings. The open-ended question on reasons respondents were not interested in attending an information meeting was coded by TB and RP. The association between interest in attending an information meeting (dependent variable) and having thought about end-of-life care (independent variable) was assessed by conducting logistic regression analyses. Also, for the people who had thought about end-of-life topics, the association between interest in attending an information meeting (dependent variable) and having discussed end-of-life topics with family/friends, with healthcare professionals, or having written down wishes (independent variables) was assessed. All regression analyses were corrected for age and sex. A p-value of < 0.05 was considered significant.

## Results

### Characteristics of study population

The study population included roughly equal numbers of men and women (Table 1). Most respondents were younger than 75 years old (66%), did not have a migration background (86.5%) and were not religious (63%). Also, 40.4% of respondents had a low level of education, 27% a middle level of education and 32.6% a high level of education. A small minority of respondents had inadequate health literacy (1.5%). Some respondents had experience with palliative care through family, friends and/or acquaintances (41.6%), and 21.5% perceived their health status as bad.

**Table 1:** Characteristics of respondents stratified by interest in attending an information meeting on end-of-life care (*N* = 1242)

	Total (*N* = 1242)	Not interested (*N* = 411)	(Possibly) interested in the future (*N* = 410)	Currently interested (*N* = 413)	Chi-square (*p* < 0.05)
	*N* (column %)	*N* (column %)	*N* (column %)	*N* (column %)	
**Demographic characteristics**					
Sex (female)	631 (50.8)	222 (54.0)	186 (45.4)	220 (53.3)	0.023
Age					**<0.001 **
65–70	406 (32.7)	130 (31.6)	162 (39.5)	111 (26.9)	
70–75	413 (33.3)	123 (29.9)	140 (34.1)	147 (35.6)	
75–80	228 (18.4)	80 (19.5)	58 (14.1)	90 (21.8)	
≥ 80	195 (15.7)	78 (19.0)	50 (12.2)	65 (15.7)	
Migration background (yes)	167 (13.5)	52 (12.8)	53 (13.0)	60 (14.6)	0.692
Religiosity (yes)	456 (37.0)	154 (37.8)	151 (37.1)	150 (36.4)	0.914
Level of education					**< 0.001**
Low	500 (40.4)	200 (48.9)	150 (36.6)	150 (36.4)	
Middle	335 (27.0)	104 (25.4)	110 (26.8)	117 (28.4)	
High	404 (32.6)	105 (25.7)	150 (36.6)	145 (35.2)	
Net household income					0.314
Below basic state pension (AOW*): ≤1,350 euro	184 (15.7)	63 (16.4)	56 (14.4)	65 (16.5)	
Between basic state pension and median income: 1,350–2,350 euro	606 (51.6)	202 (52.6)	187 (48.2)	212 (53.8)	
Between median income and 1.5 times median income: 2,350–3,850 euro	237 (20.2)	77 (20.1)	85 (21.9)	72 (18.3)	
More than 1.5 times median income: >3,850 euro	147 (12.5)	42 (10.9)	60 (15.5)	45 (11.4)	
Urbanisation					0.077
Very urban	185 (14.9)	64 (15.6)	55 (13.4)	66 (16.0)	
Strongly urban	276 (22.3)	88 (21.5)	86 (21.0)	101 (24.5)	
Moderately urban	258 (20.8)	73 (17.8)	89 (21.8)	94 (22.8)	
Not very urban	236 (19.0)	77 (18.8)	93 (22.7)	63 (15.3)	
Not urban	285 (23.0)	108 (26.3)	86 (21.0)	89 (21.5)	
**Health- and care-related characteristics**					
Self-perceived health status (bad)	267 (21.5)	98 (23.8)	74 (18.0)	94 (22.8)	0.100
Health literacy (inadequate)	19 (1.5)	11 (2.7)	5 (1.2)	3 (0.7)	0.062
Personal experience with palliative care in their environment (yes)	497 (41.6)	155 (39.9)	174 (43.5)	166 (41.1)	0.585
Frequency contact with GP per year					0.064
None	292 (23.5)	110 (26.8)	103 (25.1)	77 (18.6)	
1–4 times	819 (65.9)	256 (62.3)	266 (64.9)	291 (70.5)	
5 or more	131 (10.5)	45 (10.9)	41 (10.0)	45 (10.9)	
Frequency contact with MS per year					0.342
None	553 (44.5)	191 (46.5)	188 (45.9)	170 (41.2)	
1–4 times	581 (46.8)	191 (46.5)	183 (44.6)	203 (49.2)	
5 or more	108 (8.7)	29 (7.1)	39 (9.5)	40 (9.7)	
Trust in GPs providing good care at the end of life					0.061
No to not much trust	73 (6.3)	19 (4.9)	33 (8.7)	21 (5.3)	
Some to a lot of trust	1094 (93.7)	370 (95.1)	348 (91.3)	374 (94.7)	
Trust in GPs respecting wishes at the end of life					0.084
No to not much trust	135 (11.6)	353 (9.5)	317 (14.6)	354 (11.1)	
Some to a lot of trust	1026 (88.4)	10 (90.5)	4 (85.4)	7 (88.9)	

*Number of missing values* Interest information meetings (8), sex (0), age (0), migration background (8), religiosity (9), level of education (3), net household income (68), urbanisation (2), perceived health status (0), health literacy (10), experience with palliative care environment (46), frequency of contact with GP per year (0), frequency of contact with MS per year (0), trust in GPs providing good care at the end of life (75), trust in GPs respecting wishes at the end of life (81)

*N *Number*, GP *General practitioner*, MS *Medical specialist

*The AOW pension is a basic state pension provided by the Dutch government to people who have reached AOW pension age

### Interest in attending an information meeting

One-third of respondents reported that they would attend an information meeting if they were invited this week (33.5%), one-third (33.2%) (possibly) in the future, and one-third (33.3%) would not attend. Respondents who were not interested in attending an information meeting more often had a low level of education (*p* < 0.001). Respondents who were interested in attending an information meeting in the future were more often younger (65–70 years) (*p* < 0.001) and male (*p* = 0.023). Other characteristics were not associated with interest in information meetings.

Respondents who were currently interested in attending an information meeting reported that they want to know more about the end of life (39.7%), are thinking about the end of life (37.5%), that the subject is important to them (28.8%), and that the subject is important to their loved ones (20.6%). Respondents who were not interested in attending an information meeting reported reasons such as that they are not ready yet (e.g. too healthy or young) (31.1%), that they have already engaged in ACP (e.g. discussed and/or written down wishes) (17.5%), and that they ‘know enough’ about the topic (9.8%). A minority of the respondents reported that they prefer to discuss the topic individually (9.2%) and/or not in a public meeting (4.4%). Also, some respondents mentioned the COVID-19 pandemic as a reason not to attend (3.8%).

**Fig. 1:**
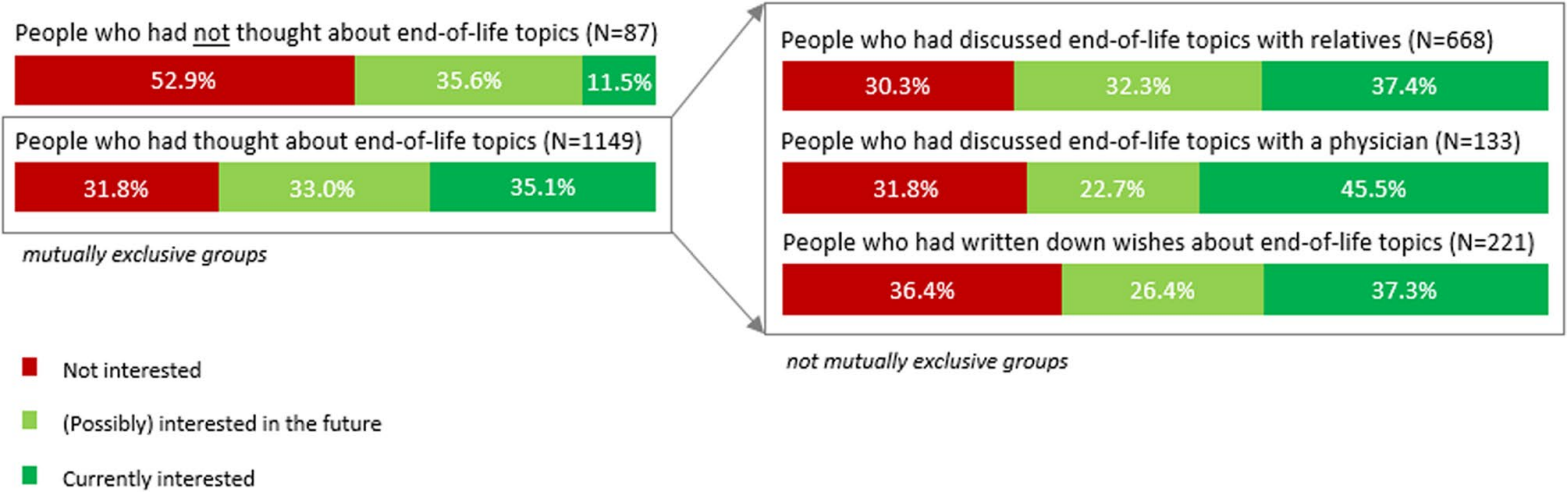
Interest in attending an information meeting stratified by previous ACP behaviour (*N* = 1242)

Figure 1 presents interest in attending an information meeting by previous ACP behaviour. Respondents who had thought about end-of-life topics (OR 2.54 CI 1.63–3.96) were significantly more likely to be currently and (possibly) interested in the future to attend an information meeting compared to respondents who had not thought about end-of-life topics. The odds ratio was larger when assessing currently interested only versus not interested (OR 5.26 CI 2.61–10.62). Regarding people who had thought about end-of-life topics, no significant association was found between being interested in information meetings and having discussed end-of-life topics with family/friends, with healthcare professionals or having written down wishes (Table 2).

**Table 2:** Association between previous ACP behaviour and interest in information meetings on end-of-life care (*N* = 1242)

	Not interested (*N* = 411)	(Possibly) interested in the future (*N* = 410)	Currently interested (*N* = 413)	Currently interested vs. not interested	(Possibly) interested in the future vs. not interested	Currently and (possibly) interested in the future vs. not interested
	*N* (row %)	*N* (row %)	*N* (row %)	OR (95% CI)	OR (95% CI)	OR (95% CI)
**Model 1**						
Having thought about end-of-life topics						
No	46 (52.9)	31 (35.6)	10 (11.5)	ref.	ref.	ref.
Yes	365 (31.8)	379 (33.0)	403 (35.1)	**5.26 (2.61–10.62)**	**1.66 (1.02–2.69)**	**2.54 (1.63–3.96)**
Model 2*						
Having discussed end-of-life topics with family/friends						
No	163 (33.9)	164 (34.1)	154 (32.0)	ref.	ref.	ref.
Yes	202 (30.3)	215 (32.3)	249 (37.4)	1.33 (0.99–1.78)	1.19 (0.88–1.61)	1.26 (0.97–1.63)
Having discussed end-of-life topics with a HCP						
No	323 (31.8)	349 (34.4)	343 (33.8)	ref.	ref.	ref.
Yes	42 (31.8)	30 (22.7)	60 (45.5)	1.39 (0.89–2.15)	0.84 (0.49–1.41)	1.12 (0.74–1.68)
Having written down wishes						
No	282 (30.7)	321 (34.6)	321 (34.6)	ref.	ref.	ref.
Yes	80 (36.4)	58 (26.4)	82 (37.3)	0.85 (0.59–1.22)	0.73 (0.49–1.08)	0.79 (0.58–1.09)

All analyses were corrected for age and sex

*Number of missing values *Interest in information meetings (8), ACP behaviour (6)

*N* Number, *OR* Odds Ratio, *CI *Confidence Interval, *ref* Reference category, *HCP* Healthcare professional

*Model 2 only includes people who had thought about end-of-life topics (*N* = 1149)

Respondents who were interested in information meetings (*N* = 823) were most interested in information on possibilities for care at home (88.1%), symptom relief (87.7%), advance directives (80.6%), support for informal caregivers (80.1%), and euthanasia (76.1%) (Table 3). People who had not thought about end-of-life topics were roughly equally interested in possibilities of care at home, symptom relief, and support for informal caregivers, but to a lesser degree in advance directives (61.0% vs. 81.6%) and euthanasia (53.7% vs. 77.2%) compared to people who had thought about end-of-life topics.

**Table 3:** Interest in information meetings stratified by previous ACP behaviour (*N* = 823)

	Total (*N* = 823)	People who did not think about end-of-life topics (*N* = 41)	People who did think about end-of-life topics (*N* = 782)
	*N* (column %)	*N* (column %)	*N* (column %)
Interest in topics for information meetings			
Possibilities for care at home	725 (88.1)	35 (85.4)	690 (88.2)
Symptom relief	722 (87.7)	36 (87.8)	686 (87.7)
Advance directives	663 (80.6)	25 (61.0)	638 (81.6)
Support for informal caregivers	659 (80.1)	33 (80.5)	626 (80.1)
Euthanasia	626 (76.1)	22 (53.7)	604 (77.2)
Care in hospice facilities	575 (69.9)	23 (56.1)	552 (70.6)
Final hours before death	545 (66.2)	21 (51.2)	524 (67.0)
Loss and grief	518 (62.9)	20 (48.8)	498 (63.7)
Care in nursing homes	496 (60.3)	22 (53.7)	474 (60.6)
Life questions, meaning and spirituality	303 (36.8)	17 (41.5)	286 (36.6)
Interest in attending an information meeting if information received through			
Personal letter from another organisation than GP (e.g. municipality)	450 (54.7)	16 (39.0)	434 (55.6)
Personal letter from my GP	421 (51.2)	18 (43.9)	403 (51.6)
Announcement in a local newspaper or folder	184 (22.4)	13 (31.7)	171 (21.9)
Poster in a healthcare facility	118 (14.4)	2 (4.9)	116 (14.9)
Announcement in a church folder and/or during a service	49 (6.0)	1 (2.4)	48 (6.1)
Poster in a library and/or community centre	41 (5.0)	2 (4.9)	39 (5.0)
Interest in attending an information meeting organised by			
Their own GP			
No	58 (7.1)	5 (12.2)	53 (6.8)
Maybe	367 (44.6)	27 (65.9)	340 (43.5)
Yes	397 (48.3)	9 (22.0)	388 (49.7)
Other healthcare facilities (e.g. hospital or residential care facility)			
No	254 (30.9)	20 (48.8)	234 (30)
Maybe	421 (51.2)	18 (43.9)	403 (51.6)
Yes	147 (17.9)	3 (7.3)	144 (18.4)
Hospice facility			
No	305 (37.1)	23 (56.1)	282 (36.1)
Maybe	381 (46.4)	16 (39.0)	365 (46.7)
Yes	136 (16.5)	2 (4.9)	134 (17.2)
Welfare organisation			
No	338 (41.1)	19 (46.3)	319 (40.8)
Maybe	390 (47.4)	19 (46.3)	371 (47.5)
Yes	94 (11.4)	3 (7.3)	91 (11.7)
Organisation with spiritual caregivers			
No	489 (59.5)	26 (63.4)	463 (59.3)
Maybe	259 (31.5)	14 (34.1)	245 (31.4)
Yes	74 (9.0)	1 (2.4)	73 (9.3)

*Topics*,* invitation and organisation of information meetings*

*Number of missing values* Interest in information meetings (8), Interest in subject for information meetings (0), Interest in information meeting if received information from (0), Interest in information meetings organised by (1)

*N* Number, *% *Percentage, *GP* General practitioner

Overall, respondents were most interested in attending an information meeting if they received an invitation or information through a personal letter from their GP (51.2%) or another organisation such as the municipality (54.7%). Also, they preferred their own GP (48.3%) or another healthcare facility such as a hospital or care home (17.9%) to organise information meetings.

## Discussion

This nationwide study among older people provides insight in the potential reach of information meetings on end-of-life care and how these meetings can best be shaped. The results show that one-third of older people would attend an information meeting on end-of-life care this week, one-third (possibly) in the future, and one-third would not attend. Older people who had already thought about end-of-life topics were more likely to be interested in attending an information meeting. Overall, older people were interested in a broad range of topics of which they were most interested in possibilities for care at home, symptom relief, and advance directives. Further, they prefer to be invited through a personal letter from, for example, their GP or another organisation such as the municipality. Also, older people preferred their GP to organise the information meeting.

### The majority of older people would attend an information meeting on end-of-life care

Our study indicates that the majority of older people is interested in attending an information meeting on end-of-life care, suggesting a large potential reach and appeal of such meetings. Attending an information meeting may help older people prepare for engagement in ACP in the future, as it may resolve misconceptions and stimulate conceptualization of wishes and preferences regarding future care. Abu et al. (2019) suggests that this conceptualization is necessary to prepare for ACP discussions with family/friends and healthcare professionals [7]. The high interest of older people in information is not surprising; systematically reviewing the literature, Sharp et al. (2013) concluded that 61 to 91% of older people (depending on i.e. country and setting) wants to discuss end-of-life care [29]. Although attending an information meeting is not the same as engaging in ACP, it does show the willingness and readiness of older people to engage in the topic.

Moreover, the questionnaire on palliative care was distributed in September 2020, during the COVID-19 pandemic. During that period, older people were advised to isolate themselves [30]. Interest in attending an information meeting *this week* might therefore be underestimated. Some respondents mentioned this as reason not to attend an information meeting.

Further, a broad and diverse group of older people is interested in attending information meetings. For example, interest in information meetings did not differ for religiosity or having a migration background. Also, health- and care-related characteristics, such as self-perceived health, were not associated with interest in information meetings; older people in good and bad health are equally interested. This supports the argument for already introducing (information on) ACP to older people who are still in good health and living independently in the community [6].

### Thinking about end-of-life care

Older people who are already thinking about end-of-life care are more likely to be interested in information meetings. An explanation may be that they are more aware of the need for and ready to engage in ACP. Still, half of the older people who had not thought about end-of-life care were interested in information meetings. This suggests that information meetings are a suitable medium to reach and inform older people with different levels of engagement in ACP. Considering that misconceptions about palliative care are very common among all older people [31], information meetings should not solely focus on reaching people who have not thought about end-of-life care, but all older people.

Notably, within the group of people who had already thought about end-of-life care, their further engagement in ACP was not associated with interest in information meetings. Engagement in ACP may potentially contribute to competing motivations for both attending and not attending an information meeting on end-of-life care. Some people mentioned that they lacked interest because, with engaging in ACP, they felt that they had ‘already taken care of it’ or had been sufficiently informed. However, it is likely that others may express interest in information meetings precisely due to their current engagement in ACP or experienced lack of knowledge (regarding specific topics) during the process of ACP. Interest in information meetings among older people with different levels of ACP engagement aligns with the consensus definition stressing that ACP is a process [3], potentially involving ongoing information needs.

### Tailoring information meetings to the preferences of older people

The interview study of Glaudemans et al. (2018) suggests that, with systematic ACP approaches (e.g. information meetings, screening), euthanasia and resuscitation are always discussed and thus considered important by GPs [5]. However, our findings show that older people are interested in a broad range of topics, not only including (how to document) end-of-life decisions (e.g. euthanasia, advance directives), but first and foremost care topics such as possibilities for care at home, symptom relief and support for informal caregivers. More specifically, older people who had not thought about end-of-life care yet were equally interested in the care topics, but somewhat less interested in end-of-life decisions, compared to older people who had thought about end-of-life care. Still, over half of the people who had not thought about end-of-life care were interested in information on end-of-life decisions. To accommodate the preferences of older people, information meetings should cover a broad range of topics.

### Involvement of GPs in information meetings

Overall, older people prefer to be invited to an information meeting through a personal invitation from their GP or another organisation such as the municipality. More general recruitment strategies (e.g. posters or newspaper announcements) were considered less appealing. Further, older people preferred their GP to organise the information meeting over any other party (i.e. hospital, hospice facility, welfare organisation). This aligns with previous research attributing a systemic role to general practice in ACP, because of the longstanding doctor-patient relationship and the related trust [32, 33]. For example, De Vleminck et al. (2018) suggests that the majority of older people would appreciate it if their GP would initiate an ACP conversation [33]. Given the considerable workload of GPs, they could consider working together with other parties in organising information meetings.

### Strengths and limitations

To our knowledge, this is the first study to explore older people’s interest in and preferences for information meetings on end-of-life care. Moreover, our study consists of a sample of older people drawn from a nationwide sample of the adult population, adding to the generalizability of conclusions. However, older people with inadequate health literacy were underrepresented. In 2021, 7.4% of Dutch older people were estimated to have inadequate health literacy [34], compared to 1.5% in our study. Also, distribution of the questionnaire during the COVID-19 pandemic might have resulted in an underestimation of older people’s interest in information meetings.

## Conclusion

This study shows that information meetings on end-of-life care are appealing to a large and diverse group of older people. Information meetings should cover a broad range of topics, ranging from possibilities of care at home and symptom relief to end-of-life decisions. For these meetings, older people prefer to be invited through a personal letter and value the involvement of their GP. Therefore, meetings should preferably be organised by or together with the GP. Information meetings on end-of-life care can serve as a low-threshold intervention to stimulate ACP in older people.

## Supplementary Information

Supplementary Material 1

## Data Availability

The data is, after registration, freely available for scientific, policy or socially relevant (i.e. non-commercial) research from the LISS data archive (see https://www.dataarchive.lissdata.nl, in English, assembled study 271), like all other studies conducted with the LISS panel.

## Abbreviations

ACP: Advance care planning
LISS: Longitudinal internet studies for the social sciences
Amsterdam UMC: Amsterdam University Medical Center
GP: General practitioner
MS: Medical specialist
