# Supplementary material for: Two-thirds of older people is interested in information meetings on end-of life care to stimulate advance care planning: a national survey
Source: BMC Geriatr. 2025 Jul 31;25:574. doi: 10.1186/s12877-025-06231-x (PMC12315270; doi:10.1186/s12877-025-06231-x)
Supplement: Supplementary file 1 — Supplementary Material 1 [file 12877_2025_6231_MOESM1_ESM.docx]

| *Appendix 1. Description of measurements of characteristics.* | | |
| --- | --- | --- |
| **Variable** | **Original** | **Recoded** |
| Interest in attending an information meeting | “Suppose you receive an invitation for an information meeting on end-of-life care this week. Would you go?” (yes/no).  When respondents answered ‘no’, they were asked ‘Do you think you would attend an information meeting on end-of-life care in the future? (yes, definitely/yes, maybe/no). | For the purpose of analyses, the two questions were categorized into [1] currently interested (answered ‘yes’ to the first question), [2] (possibly) interested in the future (answered ‘yes, definitely’ or ‘yes, maybe’ to the second question) and [3] not interested (answered ‘no’ to the first and second question). The categories ‘currently interested’ and ‘(possibly) interested in the future’ were analysed separately as well as together. |
| Advance Care Planning | Respondents were asked whether they had a) thought about, b) discussed with family/friends, c) discussed with a healthcare professional, and/or had d) written down wishes about the following topics:   - whether or not they want / can continue to live at home - would like to go to hospital - would like to be admitted to a nursing home - want to be resuscitated - which treatments they would and would not want any more in certain circumstances - would want euthanasia in certain circumstances - who could make medical decisions for them if they are no longer able to do so themselves. | First, respondents were categorised in the following (mutually exclusive) categories: people who had and had not thought about end-of-life topics. People who had thought about end-of-life care were further categorised in the following (not mutually exclusive) categories of ACP behaviour: people who [1] discussed end-of-life topics with family/friends, [2] discussed end-of-life topics with a healthcare professional, and [3] had written down wishes regarding end-of-life care (advance directive). |
| Age | Continuous | 1. 65-70 years 2. 70-75 year 3. 75-80 years 4. 80 years and older |
| Sex | Male; female | NA |
| Migration background | 1. Dutch background 2. First generation western migrant 3. First generation non-western migrant 4. Second generation western migrant 5. Second generation non-western migrant | 1. No (including Dutch background) 2. Yes (else) |
| Religiosity | Do you consider yourself to belong to a denomination or religious group?   1. No 2. Yes | NA |
| Level of education | 1. Primary education 2. Pre-vocational secondary education (VMBO) 3. Senior general secondary education (HAVO)/ pre-university education (VWO) 4. Secondary vocational education (MBO) 5. Higher professional education (HBO) 6. University education (WO) | Categorisation according to the standard categorisation of Statistics Netherlands (1):   1. Low level of education (including primary education, VMBO) 2. Middle level of education (including HAVO/VWO, MBO) 3. High level of education (including HBO, WO) |
| Net household income | (Net household income / √number of household members) categorised into quartiles | Net household income corrected for the number of household members   1. Quartile 1: 0-1529 euro 2. Quartile 2: 1534-1945 3. Quartile 3: 1945-2546 4. Quartile 4: 2547-12021 |
| Urbanisation | 1. Very urban 2. Highly urban 3. Moderately urban 4. Slightly urban 5. Not urban | NA |
| Self-perceived health status | 1. Very good 2. Good 3. Moderate 4. Bad 5. Very bad | 1. Bad (including very bad, bad, and moderate) 2. Good (including good and very good) |
| Previous experience with palliative care in their environment | Have you ever had to deal with palliative care in your environment, for example with one of your relatives (e.g. family)?   1. No 2. Yes | NA |
| Health literacy | Three questions:   1. How often does someone help you to read letters or leaflets from your GP, the hospital or other healthcare institutions? 2. How sure are you that you fill in medical forms correctly? 3. How often is it difficult for you to learn more about your health because you do not understand written information?   To question a. and c. respondents could answer never (score 4); occasionally (3); sometimes (2); often (1); always (0). To question b. respondents could answer extremely (score 4); quite a bit (3); somewhat (2); a little bit (1); not at all (0). Mean score of the three questions was calculated. According to Chew’s Set of Brief Screening Questions (2). | 1. Adequate health literacy (mean score >2) 2. Inadequate health literacy (mean score ≤2) |
| Frequency of contact with general practitioner (GP) per year | 1. None 2. 1-4 times per year 3. 5-11 times per year 4. 12 or more times per year | 1. None 2. 1-4 times per year 3. 5 or more times per year |
| Frequency of contact with medical specialist (MS) per year | 1. None 2. 1-4 times per year 3. 5-11 times per year 4. 12 or more times per year | 1. None 2. 1-4 times per year 3. 5 or more times per year |
| Trust in the GP providing good care at the end of life | 1. No trust 2. Not much trust 3. Some trust 4. A lot of trust | 1. No to not much trust 2. Some to a lot of trust |
| Trust in the GP respecting wishes at the end of life | 1. No trust 2. Not much trust 3. Some trust 4. A lot of trust | 1. No to not much trust 2. Some to a lot of trust |

**Bibliography**

1. Statistiek CBv. Standaard Onderwijsindeling 2021 (SOI 2021) Den Haag: Centraal Bureau voor Statistiek; 2022 [Available from: <https://www.cbs.nl/nl-nl/onze-diensten/methoden/begrippen/standaard-onderwijsindeling-2021--soi-2021-->.

2. Chew LD, Bradley KA, Boyko EJ. Brief questions to identify patients with inadequate health literacy. Family Medicine. 2004;36(8):588-94.
